# Engagement and Usability of a Cognitive Behavioral Therapy Mobile App Compared With Web-Based Cognitive Behavioral Therapy Among College Students: Randomized Heuristic Trial

**DOI:** 10.2196/14146

**Published:** 2020-02-03

**Authors:** Saptarshi Purkayastha, Siva Abhishek Addepally, Sherri Bucher

**Affiliations:** 1 Indiana University - Purdue University Indianapolis Indianapolis, IN United States; 2 Indiana University School of Medicine Indianapolis, IN United States

**Keywords:** cognitive behavioral therapy, mHealth, mental health, heuristics, usability

## Abstract

**Background:**

Recent evidence in mobile health has demonstrated that, in some cases, apps are an effective way to improve health care delivery. Health care interventions delivered via mobile technology have demonstrated both practicality and affordability. Lately, cognitive behavioral therapy (CBT) interventions delivered over the internet have also shown a meaningful impact on patients with anxiety and depression.

**Objective:**

Given the growing proliferation of smartphones and the trust in apps to support improved health behaviors and outcomes, we were interested in comparing a mobile app with Web-based methods for the delivery of CBT. This study aimed to compare the usability of a CBT mobile app called MoodTrainer with an evidence-based website called MoodGYM.

**Methods:**

We used convenience sampling to recruit 30 students from a large Midwestern university and randomly assigned them to either the MoodGYM or MoodTrainer user group. The trial period ran for 2 weeks, after which the students completed a self-assessment survey based on Nielsen heuristics. Statistical analysis was performed to compare the survey results from the 2 groups. We also compared the number of modules attempted or completed and the time spent on CBT strategies.

**Results:**

The results indicate that the MoodTrainer app received a higher usability score when compared with MoodGYM. Overall, 87% (13/15) of the participants felt that it was easy to navigate through the MoodTrainer app compared with 80% (12/15) of the MoodGYM participants. All MoodTrainer participants agreed that the app was easy to use and did not require any external assistance, whereas only 67% (10/15) had the same opinion for MoodGYM. Furthermore, 67% (10/15) of the MoodTrainer participants found that the navigation controls were easy to locate compared with 80% (12/15) of the MoodGYM participants. MoodTrainer users, on average, completed 2.5 modules compared with 1 module completed by MoodGYM users.

**Conclusions:**

As among the first studies to directly compare the usability of a mobile app–based CBT with smartphone-specific features against a Web-based CBT, there is an opportunity for app-based CBT as, at least in our limited trial, it was more usable and engaging. The study was limited to evaluate usability only and not the clinical effectiveness of the app.

## Introduction

### The Need for Digital Interventions in Mental Health

According to the World Health Organization, depression is a widespread chronic mental health issue, affecting over 350 million individuals globally [1]. About 16.1 million adults over the age of 18 years in the United States had at least one depressive episode in the past year [2]. Globally, between 2011 and 2030, the impact of depression on the aggregate economic output is estimated to be around US $5.36 trillion [1]. The reduction of costs related to diagnosis, management, and treatment of mental health issues, including depression, is a crucial target for global health system partners [3-6].

Prevalent methods for the management of depression include the use of both pharmacological (medication) and nonpharmacological (psychotherapy) interventions. The use of antidepressant medications is common and cost-effective; however, its clinical efficacy can be impacted by poor patient compliance to the medication regimens, which often have undesirable side effects (eg, weight gain) or take up to 6 weeks of use, upon initiation, to begin making a measurable impact on depressive symptoms [7-10]. Thus, current literature suggests supplementing pharmacological interventions with cognitive behavioral therapy (CBT) as the most effective combination treatment for the management of major depression in most patients [11,12]. Some studies have suggested that CBT, on its own, is as effective as antidepressant medications for depression [8].

Typically, a psychotherapist delivers CBT during in-person therapeutic encounters at regular intervals. During these interactions, the psychotherapist typically assesses the past and current psychological status of the client using validated instruments such as questionnaires. CBT can provide long-term protection against the relapse of depression [13], but at the same time, this is highly dependent on the skills of the psychotherapist delivering the CBT [14]. Training therapists to deliver effective CBT is expensive and can take 2 to 6 years of additional training [15]. The lack of resources to provide such training and the lack of availability of trained professionals are the most critical limiting factors restricting the widespread uptake of this type of therapy [16,17]. Thus, there is much interest in Web-based CBT as it may remove the above-mentioned barriers [18-21].

### Mobile Apps for Delivering Mental Health Interventions

Globally, health systems need effective and scalable methods that are cost-effective for delivering CBT to patients suffering from depression. Mobile health (mHealth) is rapidly gaining importance and acceptance and thus presents an exciting opportunity for delivering mental health care [22]. Recent research has found that we can attribute a user’s personality to the way they use their mobile phones [23]. This makes us wonder whether monitoring the user’s real-time data and activity [24,25] in a self-directed CBT can be a supplement to the clinical interventions as depressive adults might show unique traits in mobile phone usage. Few researchers have claimed that CBT delivered via an mHealth app is equally valid and also convenient [26,27]. By tracking the patient’s behavior using mHealth apps, health care providers can gain great insights regarding the patient’s condition and their response to psychotherapy [28]. More recently, CBT for patients with incurable cancers showed anxiety and mood improvements [29]. A systematic review by Rathbone et al on the efficacy of mHealth apps for CBT suggested collaboration, which is our starting point with the use of existing validated evidence [30]. As part of our research in this study, we developed a mobile app that included real-time user location, motion, and voice pitch tracking. However, because of the lack of accuracy of sensors across different smartphones, we had to disable those features as we have reported elsewhere [31].

## Methods

### MoodGYM—An Interactive Cognitive Behavioral Therapy Website

**Figure 1:**
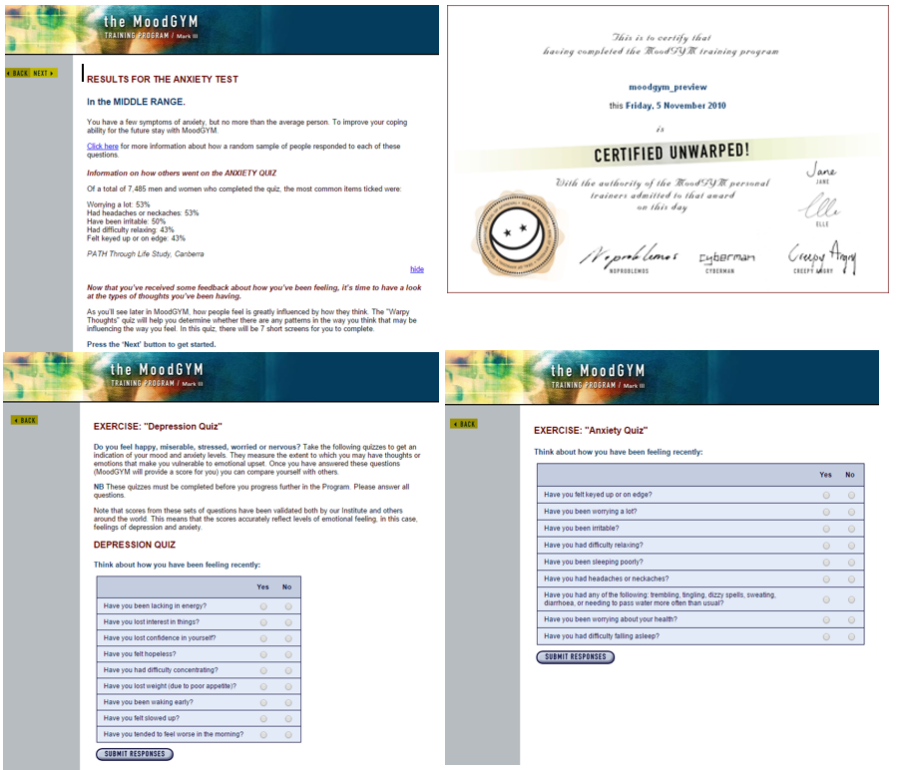
Screenshot of the MoodGYM user interface.

MoodGYM [32] is a popular, evidence-based interactive CBT website designed for the prevention, treatment, and management of depression in young people with internet access. When we conducted the study, MoodGYM was a free website, and we had access to it until March 2018. Currently, it requires an annual subscription fee for access. This website can be accessed using a computer with a standard Web browser, and it utilizes interactive and multimedia features to deliver CBT. MoodGYM requires user registration and collects basic demographics such as age group, gender, locality of residence, and the highest level of education. MoodGYM provides depression assessment scales for evaluating the progress of the user. Figure 1 contains screenshots of the version of MoodGYM used in this study.

### MoodTrainer—A Smartphone Cognitive Behavioral Therapy App

We developed a smartphone app called MoodTrainer, inspired by MoodGYM, and incorporated all the modules that were available in the earlier version of MoodGYM. The current version of MoodGYM in its terms of use describes intellectual property rights, similar to the version we used, which allow for noncommercial use. Our adaptation of the questionnaire is from published material, with appropriate references to the original authors. Before beginning the development of our app, we requested permission from the MoodGYM team to develop a mobile app for usability research purposes. We believe that the MoodGYM content was already relevant to young adults in the US context and did not need any contextual adaptation. We developed certain smartphone-specific features such as location tracking, offline storage, reminders and notifications, voice pitch and motion tracking. The voice pitch and motion tracking were not accurate as a measure of anxiety, based on the research team’s tests (see User Interface Development). So, we eventually removed these features.

#### App Development

MoodTrainer is a cross-platform, hybrid app that utilizes Web technologies, such as HTML, Cascading Style Sheets, and JavaScript, which allow us to create packages for platforms such as Android, iPhone Operating System, and Windows [33]. We used the Android package (APK) of the app for heuristic evaluation. By using the Cordova framework [34], we leveraged smartphone Application Programming Interfaces (API) such as location tracking, offline storage, reminders, and notifications, which are smartphone-specific features that are unavailable in a Web-based CBT.

#### User Interface Development

The user interface (UI) was developed in HTML5 by going through iterative cycles of prototyping, user feedback, and further development. We included a mental health expert with 8 years in practice, an informatics expert with 10 years of experience in mobile app development, and a clinically depressed student as users to provide prototyping feedback. The student was already being treated through MoodGYM and thus confirmed that it could be used in our context. Overall, we conducted 63 prototyping cycles before finalizing the version used in the heuristic evaluation. We initially used JQuery Mobile to design the app’s UI. Although the app was fast and responsive, preliminary UI testing with a small group of users made us rethink our app’s usability.

**Figure 2:**
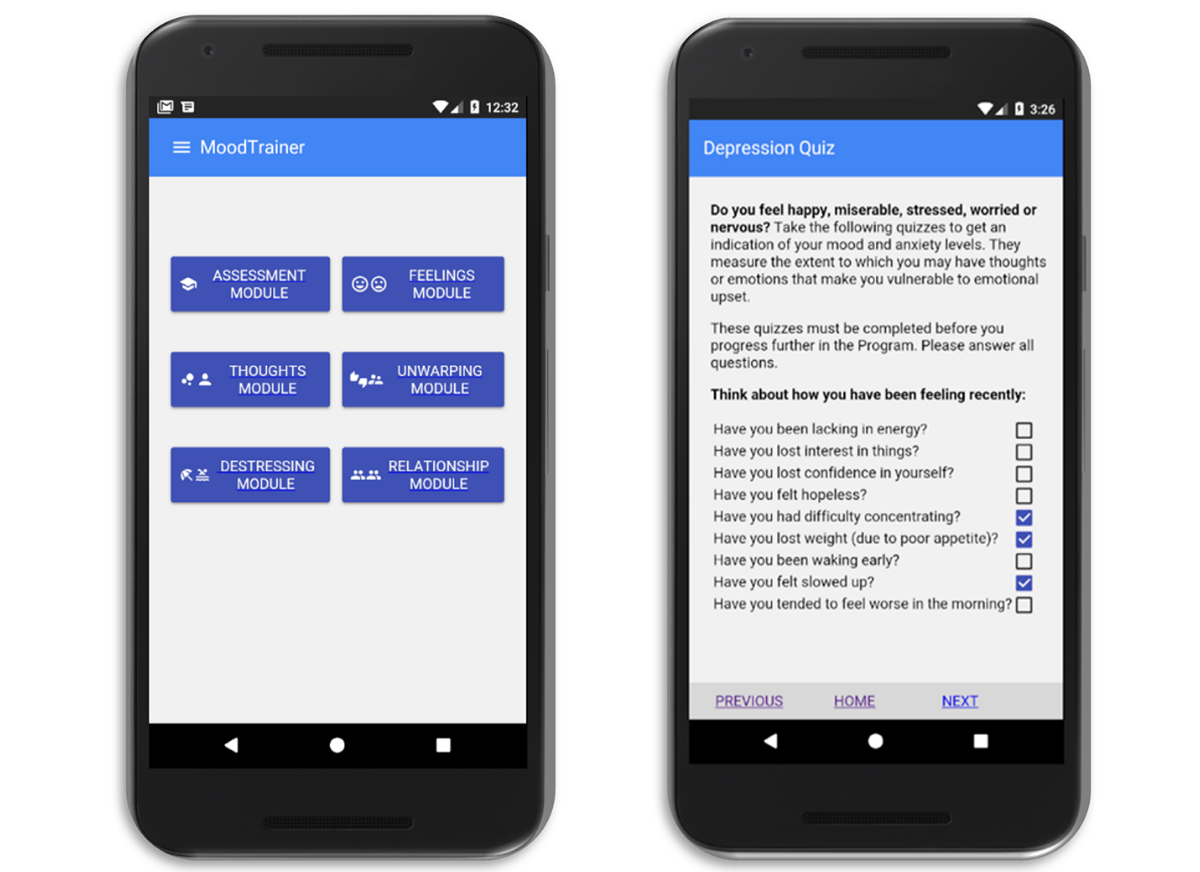
User interface of MoodTrainer.

On the basis of this early feedback, we reimplemented the app using Polymer components that use Material Design principles [35], as can be seen in Figure 2. Material Design is a design language developed by Google for a unified interface across platforms. Material Design utilizes grid-based layouts, responsive transitions, and animations, and it utilizes lights and shadows to showcase depth [36]. By using Polymer components, we got the native Android look and feel in the app and also a design language that was familiar to the users.

We added Google Analytics (GA) to track the time spent by the user on the app, the number of screens viewed per session, session duration, app crashes, and page exits. Our app’s APK was compiled using CrossWalk [35], a Cordova plugin that bundles Chromium WebView [37] along with the app to provide the app with the up-to-date features on any device. The WebView bundled by CrossWalk facilitates the use of all device APIs and enables the utilization of all the HTML5 features on older Android phones. Despite these advantages, the plugin increases the APK size.

Literature suggests that smartphone-specific features were not utilized in any of the previous apps used for the management of depression [38]. Following are the innovations that differentiate MoodTrainer from other available CBT apps:

- Tracking user location has never been implemented in a CBT app before, as far as we know. The user location is captured at 1-min intervals. The user can see the location history on a map from the user profile page. This location tracking is not shared with anyone by default but could be shared with the provider if the user allows it. Patients with depressive episodes usually spend time alone in secluded places with many risks. Our app’s location tracking feature is a reminder to the user or, when shared with a clinician, is a potential data point in decision making.
- Notifications and reminders have been combined with evidence-based modules in MoodTrainer. Users can set reminders from the app if their mood scores are lower than the baseline. We also pushed notifications to users if they did not complete a module, nudging them to go through the mood training.

### Cognitive Behavioral Therapy Delivered by MoodTrainer and MoodGYM

The CBT aspect of MoodTrainer uses 6 modules, with each module focusing on a specific type of intervention. Previously mentioned evidence from MoodGYM suggests that the sequential format of the modules helps to improve depressive mood [39]. The modules available in the app, in the order of appearance, are as follows:

- Assessment module: The primary purpose of this module is to identify the severity of depression and anxiety symptoms experienced by the users before starting the CBT program.
- Feelings module: This module has tasks which make the users aware of their feelings and how they respond to routine life events. Later, this module associates moods with feelings.
- Thoughts module: This module provides examples of different negative thoughts. Later, in a series of activities, the user is asked to identify the thoughts that they usually experience.
- Unwarping module: This module identifies the negative thoughts experienced by the user, and the evidence-based mental health survey instruments guide the user to counter these with positive thoughts.
- Destressing module: The main goal of this module is to equip the user with destressing strategies and also help the users in applying these strategies in real-life scenarios.
- Relationship module: The final module focuses on building relationships and suggests the user to look at the bright side in a relation. This module also analyzes the users’ relation with their parents and helps them in having a better relationship with their parents.

In addition to these modules, CBT also provides scales to evaluate the user’s depression and anxiety status using Goldberg Depression and Anxiety Scales [40]. These scales are designed to aid general physicians and nonpsychiatrists in detecting depression. These scales have a specificity of 91% and a sensitivity of 86% [40]. The CBT aspect also helps in identifying dysfunctional thoughts with the help of warped thoughts scale. Parslow et al developed this scale and implemented it in MoodGYM [41]. This is a 20-item scale that covers 7 different areas such as the need for approval, love, influence on others, the need to succeed, perfectionism, external requirements for happiness, and expectations of rights. Research suggests that the warped thoughts scores correlate with the levels of depression and anxiety symptoms [41]. In addition to these scales, there are other evidence-based questionnaires integrated into the CBT modules such as (1) the Life Whacks Questionnaire [42], (2) the Measure of Parenting Style [43], and (3) the Pleasant Events Schedule [44].

### Research Design

The study received ethics approval from the institutional review board of Indiana University, with protocol number 1708867512. We used a 2-arm randomized trial design, where the enrolling participants are randomized into 2 groups—the Web-based CBT group or the mobile app–based CBT group.

### Participants

The mobile app for CBT is targeted for the management of depression in adolescents and young adults. Although it might be beneficial to exactly target clinically depressed adolescents and young adults, it was not feasible for this study, as that would require Food and Drug Administration approval for 510(k), a US market approval rule, as a mobile medical app and would require the trial to be conducted under the supervision of a mental health practitioner. The student population from which we selected our participants matched the target audience age group, including the 18+ years requirement by the MoodGYM instruments. As CBT aims to improve mood and reduce anxiety, we conducted the trial during the week of final exams, which is, as shown in previous studies, usually the time when students experience anxiety for 2 to 4 weeks [45,46]. This also informed our trial study period of 2 weeks [47].

### Recruitment Strategy and Randomization

The participants in the study were recruited by convenience sampling. We approached the participants in usual congregation areas such as waiting areas, cafeterias, and designated workstations. During the initial meeting, the participants were provided with the study details and recruited if they fulfilled the inclusion criteria and consented to participate in the study. The inclusion criteria were as follows:

- The participants should be enrolled as students at Indiana University at the time of recruitment.
- The participants must be aged 18 years or above.
- The participants must own an Android smartphone.
- To mitigate risks/harm, the participants should have no history of a major depressive episode within the past 5 years.

The study participants were randomized into 2 groups, with one group receiving a link to download the app and another group receiving log-in information to the MoodGYM website. Participants were given identifiers that were used as username in MoodGYM and as GA ID in MoodTrainer. There were 15 male and 15 female subjects, with age ranging from 24 to 44 years and a median age of 32 years. All the recruited subjects were graduate students from the health informatics program and had clinical background in pharmacy, nursing, dentistry, or medicine.

### Evaluation

We collected data on the usability of MoodTrainer and MoodGYM using a heuristics survey with a 5-point Likert scale for responses based on Nielsen heuristics [48]. The heuristic characteristics which were studied are summarized in Table 1.

**Table 1:** Summary of Nielsen heuristics evaluated in the study.

Heuristics	Aspects evaluated
Visibility of system status	This category evaluates if the user can view all the modules and access the different features present in the app.
Match between the system and the real world	This heuristic evaluates if the app can perform the task which it is expected to do.
Control and freedom	This category evaluates if the user can perform different tasks on the system and if the user has performed any unwanted task was able to recover from that without any consequences.
Consistency and standards	This heuristic evaluated if all the user interface elements of the app were consistent and if the cognitive behavioral therapy aspect of the app was straightforward.
Error prevention	This heuristic evaluated if the user had experienced any of the error prevention features and whether they helped the user in preventing errors.
Recognition rather than recall	It is better to suggest what action should be performed next rather than allowing the user to think what the next action should be. This can be achieved by having all the navigation options visible to the user.
Flexibility and ease of use	This heuristic evaluated if the app’s users were able to access the app with minimal or no external assistance.
Aesthetic and minimalistic design	This heuristic evaluated if the app’s design is minimal and the functionality that supports the task and goals of that interface.
Help users recognize, diagnose, and recover from errors	Errors are a common phenomenon when a user navigates through an app or an interface, and this heuristic evaluated if the app helped the users in recognizing and recovering from errors.
Help and documentation	This is a very important aspect of any user interface. If the user has reached a point where he is no longer coherent with what is going on in the app, the user interface must provide enough information to put the user back in place.

### Procedures

We evaluated the feasibility and acceptability of the mobile CBT using a heuristic evaluation survey and evaluated the user engagement through GA in MoodTrainer and modules completed in MoodGYM. At the end of the 2 weeks, participants received an email with the heuristic evaluation that was developed using REDCap [49]. This survey included questions regarding the features of the mobile phone–based and the Web-based CBT and the self-reported usage of the interventions. The participants were also asked to submit the summarized scores of both the depression and anxiety scales present in the app using the settings page available in the app. These data included details about when the form was completed, and scores were reported anonymously to a research-hosted District Health Information Software v2 instance, a popular open-source health management information system.

## Results

### The MoodTrainer Mobile App

The MoodTrainer app was designed with a focus on improving user engagement. The app provides location-based feedback and visual feedback of the progress of the user’s CBT module. The progress visualization of MoodTrainer was developed using Chart.js, a JavaScript library for interactive visualization. On the basis of the scores generated by the CBT scales, a chart was generated to indicate the user's progress across different modules.

Providing location feedback is one of our main innovations, a feature never implemented in a CBT app before. This feature captures the location of the user using the native device API at regular time intervals and displays it to the user on a map. Google Maps API was used to generate a map and create markers of the various locations where the user had been in the past 24 hours. On the basis of this, the user will be able to get appropriate feedback if they are spending more time in an isolated place. Initially, we planned to build a motion-sensing feature using the device’s accelerometer to detect agitations or violent movements. However, integrating this feature did not work as anticipated and had to be removed from the final version of the app. MoodTrainer also provides interactive feedback to the users regarding their performance across different CBT modules. These unique features of MoodTrainer ensure that the user is constantly engaged with the app. The level of real-time feedback provided by MoodTrainer is unique and has not been implemented previously in a CBT. The literature suggests that smartphone-specific features were not utilized in any of the previous apps that were designed for the management of depression [50].

### Heuristic Evaluation of MoodTrainer and MoodGYM

The participants were given these applications for 2 weeks to use, and at the end of the 2-week period, the participants were provided with Nielsen heuristic evaluation survey. The results of the survey are as given in Table 2, which summarizes the responses of the heuristic evaluation survey respondents who responded *Yes* and Fisher exact test results for the responses.

**Table 2:** Summary of the responses and the statistics of the heuristic evaluation survey.

Heuristic and question			MoodTrainer (n=15)		MoodGYM (n=15)		*P* value (2-tailed)
**Visibility of system status**							
	Is the app home screen clearly depicting the features and modules?	15		12		.22	
	Are the buttons clear and of appropriate size to select?	14		14		>.99	
	Were you able to access the location feedback provided by the app?	12		—^a^		—	
**Match between the system and the real world**							
	Do the actions provided by the system match the actions performed by user?	15		12		.22	
	Does the activity scheduling match a real-time experience in scheduling activities?	12		11		>.99	
	Are the activities for identifying warped thoughts effective enough?	14		15		>.99	
	Are the modules effective enough in identifying positive thoughts?	14		15		>.99	
**User control and freedom**							
	Is it easy to navigate across the various modules and features present in the app?	13		12		>.99	
	Are the navigation controls easy to locate?	10		12		.68	
**Consistency and standards**							
	Are all the screens consistent in format (font, color, and layout) across the application?	12		13		>.99	
	Are all the questions in the application easy to understand and straightforward?	12		10		.68	
**Error prevention**							
	Is navigation to the next screen prevented until the current task is completed?	9		13		.21	
	Can you randomly navigate across the modules?	10		9		.68	
**Recognition rather than recall**							
	Are all the buttons used relevant?	15		12		.22	
	Are the buttons and icons used consistent and similar across the application?	14		15		>.99	
**Flexibility and efficacy of use**							
	Are the buttons navigating you to the appropriate location?	15		15		>.99	
	Is the application easy to use and navigate with minimal or no external assistance?	15		10		*.04* ^b^	
**Aesthetic and minimalist design**							
	Does the alignment of input boxes and buttons look appropriately spaced?	12		15		.22	
	Are the colors (window/button) aesthetic to look?	10		13		.38	
**Help users recognize, diagnose, and recover from errors**							
	Is the error message understandable?	12		13		>.99	
	Does the error message provide feedback with instructions?	14		12		.59	
**Help and documentation**							
	Does the button provide enough help in completing the task?	15		15		>.99	
	Do the buttons provide enough information to reflect what they are intended to do?	15		15		>.99	

^a^The location feedback is only available in MoodTrainer and not in MoodGYM.

^b^Statistically significant difference between MoodTrainer and MoodGYM.

#### Heuristic 1—Visibility of System Status

Most participants agreed that both the interventions were clear and that the UI depicts all the features and modules of the application. However, some MoodGYM users commented that the website’s home screen was not very intuitive and did not show all the features at the onset.

#### Heuristic 2—Match Between the System and the Real World

All participants agreed that both the systems performed their intended actions. Some MoodGYM participants commented that the navigation buttons were not intuitive and that they had to click a button twice to get the desired action. Furthermore, 73% (11/15) of MoodGYM participants agreed that the activity scheduling (activity in the CBT program) provided a real-time experience, whereas 86% (13/15) of the MoodTrainer users agreed that this activity matched the real-time experience. Overall, 93% (14/15) of MoodTrainer and 100% (15/15) of MoodGYM participants agreed that warped thought activities were effective. Both MoodGYM and MoodTrainer users agreed that the modules were effective in identifying positive thoughts.

#### Heuristic 3—User Control and Freedom

Overall, 13 of the 15 participants responded that it was easy to navigate across the various modules present in MoodTrainer. However, 12 of the 15 participants responded that it was easy to navigate across MoodGYM, although some of them commented that they could not locate the navigation menu, and it was difficult to navigate to the previous page as the browser back would not produce the same action. Participants had to browse through a lot of pages to find the different CBT modules. Overall, 80% (12/15) of the participants agreed that the navigation controls were easy to locate for MoodGYM, and 66% (10/15) found that the navigation controls present on the MoodTrainer app were easy to locate.

#### Heuristic 4—Consistency and Standards

Overall, 80% (12/15) of the users commented that all the screens of the MoodTrainer app were consistent with regard to font, color, and layout. One participant commented that these screens “could be more colorful and interesting” and another participant did not think that the screens were consistent. Although all MoodGYM participants agreed that the screens were consistent, some of them additionally commented that the layout could be improved for a better user experience. Furthermore, 80% (12/15) of the MoodTrainer participants agreed that all the questionnaires in the app were easy and straightforward, but some commented that they were too lengthy and required effort to understand. We believe that these validated psychological instruments are hard to understand for the uninitiated user but did not want to change for obvious clinical efficacy reasons.

#### Heuristic 5—Error Prevention

Overall, 60% (9/15) of the MoodTrainer participants and 86% (13/15) of the MoodGYM participants agreed that the navigation to the next task was prevented until the current task was completed. Furthermore, 73% (11/15) of the MoodTrainer participants agreed that they were able to navigate across different modules compared with 60% (9/15) among the MoodGYM participants.

#### Heuristic 6—Recognition Rather Than Recall

Overall, 100% (15/15) of the MoodTrainer and 80% (12/15) of the MoodGYM users agreed that the buttons were visible and understandable. Furthermore, 93% (14/15) of the MoodTrainer participants, compared with 100% (15/15) of the MoodGYM participants, agreed that the icons and other UI elements were similar across the application.

#### Heuristic 7—Flexibility and Efficacy of Use

Both MoodTrainer and MoodGYM participants agreed that the buttons were navigating them to the appropriate location. Furthermore, 100% (15/15) of the MoodTrainer participants agreed that the application was easy to use with minimal or no external assistance compared with 66% (10/15) of the MoodGYM participants.

#### Heuristic 8—Aesthetic and Minimalist Design

Overall, 80% (12/15) of the MoodTrainer users and 100% (15/15) of the MoodGYM users felt that the input boxes and buttons were appropriately placed in the UI. Furthermore, 66% (10/15) of the MoodTrainer users and 86% (10/15) of the MoodGYM users agreed that the colors, windows, and buttons were aesthetic.

#### Heuristic 9—Help Users Recognize, Diagnose, and Recover From Errors

Overall, 80% (12/15) and 86% (10/15) of MoodTrainer and MoodGYM users, respectively, felt that the error messages were understandable. Some commented that they did not encounter any errors. Furthermore, 53% (8/15) of MoodTrainer users agreed that the error messages guided them to recover from the error. A total of 2 participants mentioned that the error messages must be framed better. In addition, 73% (11/15) of the MoodGYM participants found that the error messages were helpful. Furthermore, 93% (14/15) and 80% (12/15) of the MoodTrainer and MoodGYM users, respectively, agreed that the error messages provided sufficient feedback with instructions.

#### Heuristic 10—Help and Documentation

Overall, 100% (15/15) of the MoodTrainer and MoodGYM participants agreed that there was enough information provided throughout the CBT to understand the tasks that had to be performed in the apps.

We performed Fisher exact test on the survey responses to check for statistically significant differences between the MoodGYM and MoodTrainer survey responses. It was found that easy navigation (heuristic number 7) was the only feature with a statistically significant difference. However, application usage statistics showed a larger difference between MoodGYM and MoodTrainer, highlighting a difference in user engagement.

### Application Usage Statistics

**Figure 3:**
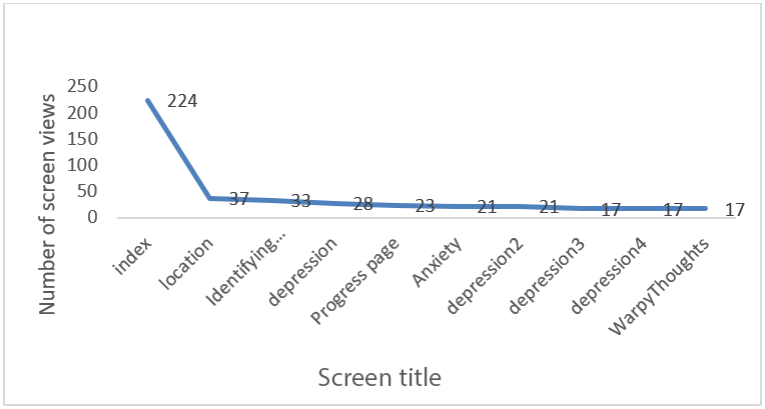
Distribution of screen views.

The app usage statistics were generated using GA can be seen in Figure 3. GA provided detailed metrics of app usage, such as the number of sessions logged, screen views, number of screens per session, and session duration. During the study period, compared to 52 in MoodGYM, a total of 67 sessions were logged by the MoodTrainer users, and there were 608 screen views during this period. On an average, the users viewed about 9 screens per session, and the average session duration was 3 min 34 seconds. During the study, it was also observed that the location feedback screen was the second most-viewed screen, with 6.0% (37/608) of the total screen views. This indicates that the study participants were interested and often looked at the location history. The progress page was among the top 5 most viewed pages during the study period, and it had about 3.8% (23/608) of the total views.

As can be seen in Table 3, 25% of the sessions lasted for about 3 min to 10 min, with an average session duration of 6 min. Furthermore, 10% of the sessions lasted between 10 min to 30 min, with an average session duration of 15 min and 24 screens per session. During the study duration, it was found that 16% of the sessions had a screen depth of 3 screens and lasted about 1 min 30 seconds min, and 11% of the sessions had a screen depth of 20+ screens and lasted about 8 min 30 seconds min. It was also observed that out of the 5 modules, on an average, MoodTrainer users completed about 2.5 modules and MoodGYM users completed only 1 module.

**Table 3:** Number of sessions by session duration for the app and web cognitive behavioral therapy.

Duration per session, seconds	MoodTrainer app sessions, n	MoodGYM web sessions, n
0-10	15	10
11-30	8	11
31-60	5	8
61-180	15	10
181-600	17	11
601-1800	7	2

## Discussion

### Differences in User Engagement Between the Two CBTs

This study is among the first to develop a mobile phone–based CBT on the basis of a Web-based CBT and compare the differences in the medium of CBT delivery. We selected MoodGYM as it is widely used in a global context. Its focus on mood disorder allowed us to apply it to the student context, where we have seen mood issues come up often. The results of our limited trial indicate that the MoodTrainer app is more usable when compared with MoodGYM. Previous studies, such as a study by Watts et al [51] in 2013, demonstrated the efficacy of delivering CBT over mobile phones. Our study affirms the computer-based versus the mobile phone–based CBT comparison for the same CBT content, with additional smartphone-specific features. More recent reviews [52] and studies [53] also show that app-based CBT is more usable and engaging. However, it is important to highlight that as more users get comfortable with smartphone interfaces and more accustomed to spending time on devices, this might simply be a reflection of user familiarization. The time spent on the app and the number of sessions logged indicate that MoodTrainer is more accepted by the users compared with MoodGYM. The usability evaluation of the app reveals that it has a user-friendly design. The app can be monotonous after a while and needs to be designed with more colors and better imagery. As we took most of the graphics and content without much change from the Web-based CBT, there is an opportunity to customize the content for delivery through mobile phones in the future. The users of the MoodTrainer app were able to access the location feedback feature. This page was also the most-viewed page during the study period. This might indicate that location tracking was useful and might improve user engagement. The statistical analysis of the survey responses showed that there were no differences in the responses between the MoodGYM and MoodTrainer users, except for a statistically significant difference in the navigation of the application. The mobile app was easier to navigate than the Web application. The session durations which were logged by the users showed that most sessions were between 3 min to 10 min. In terms of self-monitored CBT or note-taking, this may be considered usual time of daily activity. The median age of the subjects was 32 years, with the youngest at 24 years and the oldest at 44 years. So, even though our study was conducted among student population, the sample was not millennials. We also reflected that even though our subject population were not identified as depressed or having mood disorders, referring to them as mentally healthy was also not accurate. Mood disorders are fairly prevalent among college students, and many do not seek help as these are fairly temporary. Thus, we avoid the claim that our subjects are healthy, as we deliberately did not compare their clinical scales as this was only a usability study.

### Limitations

This study was restricted as a usability study and did not evaluate the clinical efficacy of the application because of cost and time constraints. The study included healthy young adults, although including young adults with depression would have given an appropriate understanding of the clinical efficacy. Depressed adults might also have cognitive issues, but there is no clear evidence on this yet. The apps were provided to users only for a brief period of 2 weeks.

### Future Work

We have attempted to make modifications based on user feedback and released the app to Google Play Store. Evaluating the app for clinical efficacy in a depressed population is the next step.

### Conclusions

We presented the first evaluation of a mobile phone–based CBT delivery, where we implemented smartphone-specific features. The study also revealed that CBT delivered by this method was more intuitive and user-friendly than Web-based CBT. This method of CBT delivery might thus be more accessible and cost-effective, with improved user engagement. Such apps being more accessible have great potential in limited-resource settings, where access to face-to-face CBT is a challenge. The MoodTrainer app does not aim to replace a clinical care provider but can supplement it as a self-supporting tool. This app might be utilized by clinical psychologists as a continuum of care to prevent the relapse of multiple episodes. It can also be used as a monitoring aid to track a patient’s progress after the therapy or during the therapy.

## Abbreviations

API: application programming interface
APK: Android package
CBT: cognitive behavioral therapy
GA: Google Analytics
mHealth: mobile health
UI: user interface

## References

[1] Bloom DE, Cafiero ET, Jané-Llopis E, Abrahams-Gessel S, Bloom LR, Fathima S, Feigl AB, Gaziano T, Mowafi M, Pandya A, Prettner K, Rosenberg L, Seligman B, Stein AZ, Weinstein C (2011). Weforum - World Economic Forum.

[2] WHO World Health Organization.

[3] Boydell KM, Hodgins M, Pignatiello A, Teshima J, Edwards H, Willis D (2014). Using technology to deliver mental health services to children and youth: a scoping review. J Can Acad Child Adolesc Psychiatry.

[4] Torio CM, Encinosa W, Berdahl T, McCormick MC, Simpson LA (2015). Annual report on health care for children and youth in the United States: national estimates of cost, utilization and expenditures for children with mental health conditions. Acad Pediatr.

[5] Donker T, Blankers M, Hedman E, Ljótsson B, Petrie K, Christensen H (2015). Economic evaluations of internet interventions for mental health: a systematic review. Psychol Med.

[6] Thornicroft G, Mehta N, Clement S, Evans-Lacko S, Doherty M, Rose D, Koschorke M, Shidhaye R, O'Reilly C, Henderson C (2016). Evidence for effective interventions to reduce mental-health-related stigma and discrimination. Lancet.

[7] Penn E, Tracy DK (2012). The drugs don't work? Antidepressants and the current and future pharmacological management of depression. Ther Adv Psychopharmacol.

[8] Amick HR, Gartlehner G, Gaynes BN, Forneris C, Asher GN, Morgan LC, Coker-Schwimmer E, Boland E, Lux LJ, Gaylord S, Bann C, Pierl CB, Lohr KN (2015). Comparative benefits and harms of second generation antidepressants and cognitive behavioral therapies in initial treatment of major depressive disorder: systematic review and meta-analysis. Br Med J.

[9] Bschor T, Kilarski LL (2016). Are antidepressants effective? A debate on their efficacy for the treatment of major depression in adults. Expert Rev Neurother.

[10] Wang S, Han C, Lee S, Jun T, Patkar AA, Masand PS, Pae C (2018). Efficacy of antidepressants: bias in randomized clinical trials and related issues. Expert Rev Clin Pharmacol.

[11] So M, Yamaguchi S, Hashimoto S, Sado M, Furukawa TA, McCrone P (2013). Is computerised CBT really helpful for adult depression?-A meta-analytic re-evaluation of CCBT for adult depression in terms of clinical implementation and methodological validity. BMC Psychiatry.

[12] Koffel E, Kuhn E, Petsoulis N, Erbes CR, Anders S, Hoffman JE, Ruzek JI, Polusny MA (2018). A randomized controlled pilot study of CBT-I Coach: feasibility, acceptability, and potential impact of a mobile phone application for patients in cognitive behavioral therapy for insomnia. Health Informatics J.

[13] Kodal A, Fjermestad K, Bjelland I, Gjestad R, Öst LG, Bjaastad JF, Haugland BS, Havik OE, Heiervang E, Wergeland GJ (2018). Long-term effectiveness of cognitive behavioral therapy for youth with anxiety disorders. J Anxiety Disord.

[14] Segal ZV, Bizzini L, Bondolfi G (2005). Cognitive behaviour therapy reduces long term risk of relapse in recurrent major depressive disorder. Evid Based Ment Health.

[15] Ferguson S, Harper S, Platz S, Sloan G, Smith K (2016). Developing specialist CBT supervision training in Scotland using blended learning: challenges and opportunities. Cogn Behav Ther.

[16] Rakovshik SG, McManus F, Vazquez-Montes M, Muse K, Ougrin D (2016). Is supervision necessary? Examining the effects of internet-based CBT training with and without supervision. J Consult Clin Psychol.

[17] Maguire N, Grellier B, Clayton K (2017). The impact of CBT training and supervision on burnout, confidence and negative beliefs in a staff group working with homeless people. Behav Cogn Psychother.

[18] Kingston D, Janes-Kelley S, Tyrrell J, Clark L, Hamza D, Holmes P, Parkes C, Moyo N, McDonald S, Austin M (2015). An integrated web-based mental health intervention of assessment-referral-care to reduce stress, anxiety, and depression in hospitalized pregnant women with medically high-risk pregnancies: a feasibility study protocol of hospital-based implementation. JMIR Res Protoc.

[19] Karyotaki E, Kleiboer A, Smit F, Turner DT, Pastor AM, Andersson G, Berger T, Botella C, Breton JM, Carlbring P, Christensen H, de Graaf E, Griffiths K, Donker T, Farrer L, Huibers MJ, Lenndin J, Mackinnon A, Meyer B, Moritz S, Riper H, Spek V, Vernmark K, Cuijpers P (2015). Predictors of treatment dropout in self-guided web-based interventions for depression: an 'individual patient data' meta-analysis. Psychol Med.

[20] Morris RR, Schueller SM, Picard RW (2015). Efficacy of a web-based, crowdsourced peer-to-peer cognitive reappraisal platform for depression: randomized controlled trial. J Med Internet Res.

[21] Saini J, Owen A, Hammer A, Brown W, Kulshreshtha A (2018). Zooming towards better health: participant experience in a web-based group cognitive behavioral intervention. iproc.

[22] Kazdin AE, Blase SL (2011). Rebooting psychotherapy research and practice to reduce the burden of mental illness. Perspect Psychol Sci.

[23] de Montjoye YA, Quoidbach J, Robic F, Pentland AS (2013). Predicting Personality Using Novel Mobile Phone-based Metrics. Proceedings of the 6th international conference on Social Computing, Behavioral-Cultural Modeling and Prediction.

[24] Donker T, Petrie K, Proudfoot J, Clarke J, Birch M, Christensen H (2013). Smartphones for smarter delivery of mental health programs: a systematic review. J Med Internet Res.

[25] Mechael P, Nemser B, Cosmaciuc R, Cole-Lewis H, Ohemeng-Dapaah S, Dusabe S, Kaonga NN, Namakula P, Shemsanga M, Burbach R, Kanter AS (2012). Capitalizing on the characteristics of mHealth to evaluate its impact. J Health Commun.

[26] Price M, Yuen EK, Goetter EM, Herbert JD, Forman EM, Acierno R, Ruggiero KJ (2014). mHealth: a mechanism to deliver more accessible, more effective mental health care. Clin Psychol Psychother.

[27] Sumsion T (2006). Client-centered Practice In Occupational Therapy: A Guide To Implementation.

[28] Greene J, Hibbard JH, Sacks R, Overton V, Parrotta CD (2015). When patient activation levels change, health outcomes and costs change, too. Health Aff (Millwood).

[29] Greer JA, Jacobs J, Pensak N, MacDonald JJ, Fuh C, Perez GK, Ward A, Tallen C, Muzikansky A, Traeger L, Penedo FJ, El-Jawahri A, Safren SA, Pirl WF, Temel JS (2019). Randomized trial of a tailored cognitive-behavioral therapy mobile application for anxiety in patients with incurable cancer. Oncologist.

[30] Rathbone AL, Clarry L, Prescott J (2017). Assessing the efficacy of mobile health apps using the basic principles of cognitive behavioral therapy: systematic review. J Med Internet Res.

[31] Addepally SA, Purkayastha S (2017). Mobile-Application Based Cognitive Behavior Therapy (CBT) for Identifying and Managing Depression and Anxiety. Proceedings of the International Conference on Digital Human Modeling and Applications in Health, Safety, Ergonomics and Risk Management.

[32] MoodGYM CBT training program.

[33] Apache Cordova.

[34] Apache Cordova.

[35] Polymer Project.

[36] Android Developers.

[37] GitHub.

[38] Singh K, Drouin K, Newmark LP, Lee J, Faxvaag A, Rozenblum R, Pabo EA, Landman A, Klinger E, Bates DW (2016). Many mobile health apps target high-need, high-cost populations, but gaps remain. Health Aff (Millwood).

[39] Christensen H, Griffiths KM, Korten AE, Brittliffe K, Groves C (2004). A comparison of changes in anxiety and depression symptoms of spontaneous users and trial participants of a cognitive behavior therapy website. J Med Internet Res.

[40] Goldberg D, Bridges K, Duncan-Jones P, Grayson D (1988). Detecting anxiety and depression in general medical settings. Br Med J.

[41] Parslow RA, Christensen H, Griffiths KM, Groves C (2006). The warpy thoughts scale: a new 20-item instrument to measure dysfunctional attitudes. Cogn Behav Ther.

[42] Tennant C, Andrews G (1976). A scale to measure the stress of life events. Aust N Z J Psychiatry.

[43] Parker G, Roussos J, Hadzi-Pavlovic D, Mitchell P, Wilhelm K, Austin MP (1997). The development of a refined measure of dysfunctional parenting and assessment of its relevance in patients with affective disorders. Psychol Med.

[44] MacPhillamy DJ, Lewinsohn PM (1982). The pleasant events schedule: studies on reliability, validity, and scale intercorrelation. J Consult Clin Psychol.

[45] Allen GJ, Giat C, Cherney RJ (1974). Locus of control, test anxiety, and student performance in a personalized instruction course. J Educ Psychol.

[46] Keeley J, Zayac R, Correia C (2008). Curvilinear relationships between statistics anxiety and performance among undergraduate students: evidence for optimal anxiety. Stat Educ Res J.

[47] Farrer LM, Gulliver A, Bennett K, Fassnacht DB, Griffiths KM (2016). Demographic and psychosocial predictors of major depression and generalised anxiety disorder in Australian university students. BMC Psychiatry.

[48] Nielsen J, Mack Rl (1994). Usability Inspection Methods.

[49] REDCap.

[50] Martínez-Pérez B, de la Torre-Díez I, López-Coronado M (2013). Mobile health applications for the most prevalent conditions by the World Health Organization: review and analysis. J Med Internet Res.

[51] Watts S, Mackenzie A, Thomas C, Griskaitis A, Mewton L, Williams A, Andrews G (2013). CBT for depression: a pilot RCT comparing mobile phone vs computer. BMC Psychiatry.

[52] Lan A, Lee A, Munroe K, McRae C, Kaleis L, Keshavjee K, Guergachi A (2018). Review of cognitive behavioural therapy mobile apps using a reference architecture embedded in the patient-provider relationship. Biomed Eng Online.

[53] Jarzabek S, Cheong K, Lim Y, Wong J, Kayanoth R, Teng J (2018). CBT Assistant Platform: web/mobile co-design solution for cognitive behavioural therapy. J Hosp Manag Health Policy.

